# Modelling approach to simulate reductions in LDL cholesterol levels after combined intake of statins and phytosterols/-stanols in humans

**DOI:** 10.1186/1476-511X-10-187

**Published:** 2011-10-21

**Authors:** Simone RBM Eussen, Cathy JM Rompelberg, Olaf H Klungel, Jan CH van Eijkeren

**Affiliations:** 1 Division of Pharmacoepidemiology and Clinical Pharmacology, Utrecht Institute for Pharmaceutical Sciences, Utrecht University, P.O. Box 80082, 3508 TB Utrecht, The Netherlands; 2 National Institute for Public Health and the Environment, P.O. Box 1, 3720 BA Bilthoven, The Netherlands

**Keywords:** model, statins, phytosterols, phytostanols, LDL cholesterol, lipids, coronary heart disease

## Abstract

**Background:**

To examine the effects on LDL cholesterol of the combined use of statins and phytosterols/-stanols, in vivo studies and clinical trials are necessary. However, for a better interpretation of the experimental data as well as to possibly predict cholesterol levels given a certain dosing regimen of statins and phytosterols/-stanols a more theoretically based approach is helpful. This study aims to construct a mathematical model to simulate reductions in low-density lipoprotein (LDL) cholesterol in persons who combine the use of statins with a high intake of phytosterols/-stanols, e.g. by the use of functional foods.

**Methods and Results:**

The proposed model includes the cholesterol pool size in the liver and serum levels of very low-density lipoprotein (VLDL) cholesterol. Both an additional and a multiplicative effect of phytosterol/-stanol intake on LDL cholesterol reduction were predicted from the model. The additional effect relates to the decrease of dietary cholesterol uptake reduction, the multiplicative effect relates to the decrease in enterohepatic recycling efficiency, causing increased cholesterol elimination through bile. From the model, it was demonstrated that a daily intake of 2 g phytosterols/-stanols reduces LDL cholesterol level by about 8% to 9% on top of the reduction resulting from statin use. The additional decrease in LDL cholesterol caused by phytosterol/-stanol use at the recommended level of 2 g/d appeared to be similar or even greater than the decrease achieved by doubling the statin dose.

**Conclusion:**

We proposed a simplified mathematical model to simulate the reduction in LDL cholesterol after separate and combined intake of statins and functional foods acting on intestinal (re)absorption of cholesterol or bile acids in humans. In future work, this model can be extended to include more complex (regulatory) mechanisms.

## Background

Increased total cholesterol and low-density lipoprotein (LDL) cholesterol levels represent a major risk for atherosclerosis and coronary heart disease (CHD). Lipid-lowering drugs, of which the hydroxymethylglutaryl-coenzyme A (HMG-CoA) reductase inhibitors (statins) have shown to be the most effective, reduce morbidity and mortality in patients with CHD [1-3]. Since the last decade of the 20^th ^century, more interest has been given to changing dietary habits, for example with the appearance of the so-called functional foods. Dairy products enriched with phytosterols/-stanols are one of the best known and most used functional foods to lower elevated total and LDL cholesterol levels. Phytosterols/-stanols are thought to compete with cholesterol for solubilisation into mixed micelles, the transport vehicles for cholesterol across the intestinal wall. Consequently, the intestinal (re)absorption of cholesterol is reduced, faecal output is increased and total and LDL cholesterol levels are lowered by 6% and 10%, respectively [4,5]. Due to the rising public awareness of health and nutritional improvement, and the mounting evidence of the effectiveness of phytosterols/-stanols, it is conceivable that in the near future an increasing number of people will combine their statin therapy with these functional foods.

To examine the effects on total and LDL cholesterol levels of the combined intake of statins and phytosterols/-stanols, in vivo studies and clinical trials are necessary. However, for a better interpretation of the experimental data as well as to possibly predict cholesterol levels given a certain dosing regimen of statins and phytosterols/-stanols a more theoretically based approach is helpful.

The present study focuses on the combined effect of atorvastatin and phytosterols/-stanols. However, our model can easily be applied to other statins and similar acting functional foods (e.g. soluble dietary fibres) as well. Moreover, based on certain genetic variants associated with cholesterol absorption and production an individual's specific reduction in total and LDL cholesterol can be predicted.

## Methods

**Figure 1:**
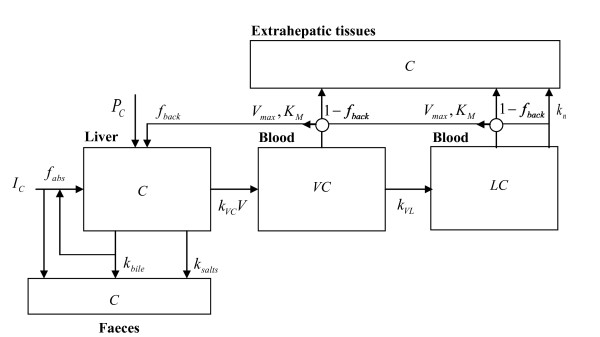
**Simplified scheme of LDL cholesterol metabolism in humans**. For detailed description of the model see text. The definition of the model variables are summarised in Table 1.

We propose a simplified mathematical model to estimate reductions in LDL cholesterol after separate and combined intake of statins and phytosterols/-stanols (Figure 1). A list of model variables and abbreviations is presented in Table 1. Since LDL is the product of very low-density lipoprotein (VLDL) delipidation and VLDL also transports, although to a lesser extent than endogenous triglycerides,[6] cholesterol from the liver to the blood circulation, our model includes the modelling of the metabolism of VLDL cholesterol as well. Also a hepatic cholesterol pool is accounted for in the model, because VLDL cholesterol secretion depends on cholesterol pool size. In the next section, we first describe a basic model which includes the modelling of the cholesterol pool, VLDL cholesterol and LDL cholesterol. Subsequently, this basic model is reformulated to express reductions in LDL cholesterol level dependent of statin and/or phytosterol/-stanol intake. Published scientific literature was used to estimate specific model parameters. In the second part of the present study (Results section), we tested the appropriateness of our model using available published experimental data.

**Table 1:** Model variables and abbreviations used in the study

Model variable	Abbreviation
Endogenously produced cholesterol	*P_C_*
Dietary cholesterol intake	*I_C_*
External daily statin dose	*S*
External daily free phytosterol/-stanol dose	*PS*
(Steady state) concentration of free cholesterol in the liver	*C*
VLDL particles	*V*
Absorbed cholesterol fraction	*f_abs_*
Fraction of produced VLDL cholesterol that re-enters the liver	*f_back_*
Association rate of VLDL particles and free cholesterol to VLDL cholesterol	*k_VC_*
Excretion of cholesterol from the cholesterol pool by bile	*k_bile_*
Excretion of cholesterol through the formation of bile salts	*k_salts_*
Reduction in cholesterol pool size	*R_C_*
Cholesterol pool concentration in absence of statins and phytosterols/-stanols	*C*_0_
Endogenous produced cholesterol in absence of statins and phytosterols/-stanols	*P*_*C*,0_
Uptake of dietary cholesterol in absence of statins and phytosterols/-stanols	*U*_*C*,0_
Absorbed cholesterol fraction in absence of statins and phytosterols/-stanols	*f_abs_*,_0_
Reduction in endogenous cholesterol production	*R_P _*
Reduction in fraction of cholesterol uptake from the diet	*R_U_*
Ratio of exponential rates of different cholesterol elimination routes	*ρ_k_*
VLDL cholesterol production rate	*P_VC _*
Transformation rate of VLDL cholesterol to LDL cholesterol	*k_VL_*
(Steady state) VLDL cholesterol concentration	*VC*
Maximum rate of change in (V)LDL cholesterol due to saturated uptake process	*V_max_*
Michaelis-Menten constant in (V)LDL cholesterol model	*K_M_*
(Steady state) LDL cholesterol concentration	*LC*
LDL cholesterol production from VLDL cholesterol	*P_LC_*
Clearance rate of LDL cholesterol through non-saturated process	*k_n_*
Maximal achievable reduction in endogenous cholesterol production	*R_P, max_*
Half maximum reduction statin dose	*S*_*P*, 1/2_
Maximal achievable reduction in fraction of cholesterol uptake from the diet	*R*_*U, max *_
Half maximum reduction phytosterol/-stanol dose	*PS*_*U*, 1/2_

### Basic cholesterol model

#### Modelling of the cholesterol pool

A mass balance is considered with cholesterol input from endogenously produced cholesterol *P_C _*and from cholesterol taken up from the diet, *I_C_*. Only a fraction *f_abs _*of dietary cholesterol is assumed to be taken up across the gut wall, and consequently the uptake of dietary cholesterol is *U_C _*= *f_abs_·I_C_*. The mass balance output consists of produced VLDL cholesterol, cholesterol cleared by elimination of excess cholesterol through bile and cholesterol needed to produce bile salts. For simplicity, we neglect the reverse cholesterol transport mediated by high-density lipoprotein (HDL) and the existence of a hepatic cholesteryl ester pool that might be involved in cholesterol homeostasis. Moreover, up- and down-regulation of LDL receptors is not considered.

The model considers only steady state levels of cholesterol, VLDL cholesterol and LDL cholesterol. For a steady cholesterol level, the input of cholesterol should be balanced by its output. Clearance of cholesterol by VLDL cholesterol formation, bile excretion and bile salts formation is assumed to be non-saturated and is described by the product of clearance rates and steady cholesterol level.

Of the daily amount of VLDL cholesterol formation, *k_VC_V *·C, the product of steady cholesterol level *C *with VLDL particles *V *and association rate *k_VC_*, a fraction *f_back _*is reabsorbed into the liver. It consists of VLDL cholesterol that is not used for LDL cholesterol production and of LDL cholesterol. The other fraction 1- *f_back _*is taken up by the extrahepatic tissues, of which part is excreted through HDL cholesterol, which will not be considered in this modelling approach. As a consequence of this recycling, the effective clearance rate of cholesterol to VLDL cholesterol is (1- *f_back_*)·*k_VC_V*. The amount of cholesterol eliminated through bile salts formation is *k_salt_·C*.

Likewise, because it is assumed that not only dietary cholesterol but also cholesterol cleared by bile with a daily amount of *k_bile_·C *is reabsorbed through enterohepatic recycling, the effective clearance rate of cholesterol through bile is (1- *f_abs_*)·*k_bile_*.

As we consider the effect of statins and phytosterols/-stanols on LDL cholesterol levels, the model becomes slightly more complicated. First, it is assumed that reduced cholesterol production *P_C _*is related to the external daily dose *S *of statins, *P_C _= P_C_*(*S*). Second, it is assumed that the reduced cholesterol fraction absorbed from dietary cholesterol intake relates to the amount of intake of phytosterols/-stanols (PS), *f_abs _*= *f_abs_*(*PS*).

In the steady state the input cholesterol *P_C_*(*S*) + *f_abs_*(*PS*)·*I_C _*is balanced by cleared cholesterol, which is the product of the effective clearance rates and the steady cholesterol level((1- *f_back_*) *k_VC_V*) + (1- *f_abs_*(*PS*)) *k_bile_+k_salts_*)·C(*S, PS*). Thus, the steady cholesterol level is:

(1) $$C\left(S,PS\right)=\frac{P_{C}\left(S\right)+f_{abs}\left(PS\right)\cdot I_{C}}{\left(1-f_{back}\right)\cdot k_{VC}V+\left(1-f_{abs}\left(PS\right)\right)\cdot k_{bile}+k_{salts}}$$

It should be noted that it is implicitly assumed that there is no interaction between statins and phytosterols/-stanols consumed, i.e. both compounds work simultaneously, independent of each other.

#### Modelling of VLDL cholesterol level

In the modelling of the cholesterol pool (equation (1)) it is assumed that the production of VLDL cholesterol *P_VC _*is proportional to both the concentration of VLDL particles and the free cholesterol level: *P_VC _= k_VC_V *·C(*S, PS*). Like for cholesterol, a steady state level *V*C(*S, PS*) of VLDL cholesterol follows from the balance between its production and its clearance. VLDL cholesterol is assumed to be cleared due to the production of LDL cholesterol with daily clearance of *k_VL_·V*C(*S, PS*) and due to saturated receptor-mediated uptake from blood into the liver and extrahepatic tissues [6]. Receptor-mediated uptake is assumed to follow Michaelis-Menten kinetics with a maximum clearance rate *V_max _*and a saturation constant *K_M_*.

Therefore, a steady state VLDL cholesterol level leads to the following mass balance for LDL cholesterol:

(2) $$P_{VC}=k_{VC}V\cdot C\left(S,PS\right)=k_{VL}\cdot VC\left(S,PS\right)+\frac{V_{max}\cdot VC\left(S,PS\right)}{K_{M}+VC\left(S,PS\right)}$$

The steady state VLDL cholesterol level can be obtained by solving the implicit equation for *V*C(*S, PS*). The explicit expression for *VC *is deduced in **Appendix 1**. Note that of the Michaelis-Menten saturated clearance of VLDL cholesterol from blood a fraction *f_back _*goes into the liver. The complementary fraction 1- *f_back _*goes into extrahepatic tissues (Figure 1).

#### Modelling of LDL cholesterol level

In the modelling of the VLDL cholesterol level (equation (2)), we assumed that the production of LDL cholesterol *P_LC _*is proportional to the steady VLDL cholesterol level: *P_LC _= k_VL_·V*C(*S, PS*). LDL cholesterol is assumed to be cleared with rate *k_n _*through a non-saturated process and by saturated uptake from blood into the liver and extrahepatic tissues by the same receptors as for the saturated uptake of VLDL cholesterol [6]. Hence, the mass balance for steady state LDL cholesterol is:

(3) $$P_{LC}=k_{VL}VC\left(S,PS\right)=k_{n}\cdot LC\left(S,PS\right)+\frac{V_{max}\cdot LC\left(S,PS\right)}{K_{M}+LC\left(S,PS\right)}$$

from which the LDL cholesterol level can be obtained by solving the implicit equation (3) for *LC*(*S, PS*). The explicit expression for *LC *can be found in **Appendix 1**. Similar as for VLDL cholesterol, a fraction *f_back _*of the Michaelis-Menten saturated clearance of LDL cholesterol from blood goes into the liver. The complementary fraction 1- *f_back _*goes into extrahepatic tissues (Figure 1). *V_max_, K_M _*are the same maximum elimination rate and saturation constant of the Michaelis-Menten saturated uptake of VLDL cholesterol from blood into the liver. These constants are assumed to be the same, but this assumption is not essential.

### Cholesterol reduction model

#### Modelling cholesterol reduction by statins and phytosterols/-stanols

When it is assumed that statins and phytosterols/-stanols work independently of each other, the reduction in the cholesterol pool size can be expressed in terms of a reduction:*R_P _*(*S*) = *P_C _*(*S*)/*P*_*C*,0 _in cholesterol production caused by statin use and a reduction: *R_U _*(*PS*) = *f_abs_*(*PS*)/*f*_*abs*,0 _in dietary cholesterol absorption across the gut wall caused by phytosterol/-stanol use. *P*_*C*, 0 _and *f*_*abs*,0 _denote the cholesterol production rate and absorption fraction in absence of statins or phytosterols/-stanols. From equation (1) it is derived in **Appendix 2 **that the corresponding reduction *R_C_*(*S, PS*) in the steady state cholesterol level is the product of the reduction due to a decrease in enterohepatic efficiency (first factor at the right side of equation (4)) and a weighted mean of the reduction due to a decrease in cholesterol production and uptake (second factor at the right side of equation (4)):

(4) $$R_{c}\left(S,PS\right)=\frac{C\left(S,PS\right)}{C_{0}}=\frac{\rho_{k}+1-{f_{abs}}_{,0}}{\underset{\text{first}\;\text{factor}}{\underset{︸}{\rho_{k}+1-f_{abs}\left(PS\right)}}}\times \underset{\text{second}\;\text{factor}}{\underset{︸}{\left(\frac{\overset{\text{first}\;\text{term}}{\overset{︷}{R_{p}\left(S\right)}}}{1+U_{C,0}/P_{C,0}}+\overset{\text{second}\;\text{term}}{\overset{︷}{\frac{R_{U}\left(S\right)}{1+P_{C,0}/U_{C,0}}}}\right)}}$$

Here, like for cholesterol production and absorption, *C*_0 _and *U*_*C*, 0 _denote, respectively, the cholesterol pool concentration and dietary uptake in absence of statins and phytosterols/-stanols. In the first factor at the right side, the ratio *ρ_k _*denotes the proportion of cholesterol elimination through VLDL cholesterol production and bile salts production to cholesterol elimination through bile excretion, as introduced in **Appendix 2**.

The following remarks should be made regarding this model. First, the effectiveness of statins or phytosterols/-stanols to lower cholesterol production is determined by the ratio of the contribution of endogenous produced cholesterol and the contribution of dietary cholesterol uptake to the cholesterol pool. Thus, when dietary cholesterol uptake is increased, the effectiveness of statins (first term in the second factor) is reduced with respect to the effectiveness of phytosterols/-stanols. Obviously, the opposite holds true for the effectiveness of phytosterols/-stanols.

Second, the reduction in the absorbed fraction of cholesterol has an additional effect in total cholesterol pool reduction (the second term in the second factor at the right side of equation (4)) and a multiplicative one (first factor at the right side of equation (4)). The additional effect relates to the decrease of dietary cholesterol uptake reduction, whereas the multiplicative effect relates to the decrease in enterohepatic recycling efficiency, causing increased cholesterol elimination through bile.

Third, the additional reduction caused by statin and phystosterol/-stanol use is a weighted sum of the reduction in cholesterol production and cholesterol absorption because 1/(1+ *P*_*C*, 0_/*U*_*C*, 0_) +1/(1+ *U*_*C*, 0_/*P*_*C*, 0_) = 1.

#### Formulating reduction as a Michaelis-Menten process

**Figure 2:**
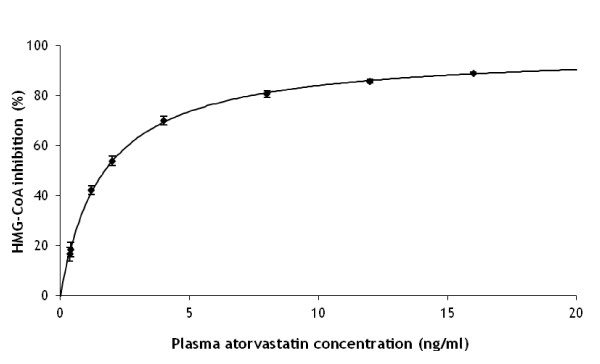
**Simulated dose-response relation between *in vitro *plasma atorvastatin concentration (ng/ml) and hydroxymethylglutaryl-coenzyme A (HMG-CoA) inhibition (%) in humans**. Maximum inhibition is 97.8% and the concentration at half maximum inhibition is 1.64 (ng/ml). Solid symbols present data by Shum et al. [7]

In the model described above, we aim to associate reductions in the cholesterol pool size to reductions in LDL cholesterol level. In order to estimate the reduction in the cholesterol pool size following statin intake, *R_P_*(*S*), a reduction model has to be assumed. From experimental *in vitro *data from Shum *et al *[7]. a reasonable model assumption is obtained as follows. Shum *et al*. related the concentration of atorvastatin in plasma to the inhibition of the enzyme HMG-CoA reductase *in vitro*. We assumed a Michaelis-Menten saturated inhibition process. This model was fitted to their experimental data and provided a nearly perfect fit (Figure 2).

From this experimental *in vitro *result, it is proposed that the reduction in endogenously produced cholesterol is most likely Michaelis-Menten saturated with administered statin dose as well:

(5) $$R_{P}\left(S\right)=1-\frac{R_{P,max}\cdot S}{S_{P,1∕2}+S}$$

where *R*_*P, max *_≤ 1 determines the maximum achievable reduction and *S*_*P*, 1/2 _is the half maximum reduction statin dose.

Based on the fact that cholesterol uptake is receptor-mediated,[8] we assumed that the reduction in the fraction absorbed cholesterol is like:

(6) $$R_{U}\left(PS\right)=1-\frac{R_{U,max}\cdot PS}{PS_{U,1∕2}+PS}$$

where *R*_*U, max *_≤ 1 determines the maximum achievable reduction and *PS*_*U*, 1/2 _is the half maximum reduction phytosterol/-stanol dose.

Thus, the total reduction in cholesterol pool size after combined intake of statins and phytosterols/-stanols is obtained by substituting equations (5) and (6) in equation (4):

(7) $$\begin{array}{ccc} R_{C}\left(S,PS\right) & =\frac{\rho_{k}+1-f_{abs,0}}{\rho_{k}+1-\left(1-R_{U,max}\cdot PS∕\left(PS_{U,1∕2}+PS\right)\right)\cdot f_{abs,0}} & \\ & \;\times \left(1-\frac{P_{C,0}}{P_{C,0}+U_{C,0}}\cdot \frac{R_{P,max}\cdot S}{S_{P,1∕2}+S}-\frac{U_{C,0}}{P_{C,0}+U_{C,0}}\cdot \frac{R_{U,max}\cdot PS}{PS_{U,1∕2}+PS}\right) \end{array}$$

In this derivation we used 1/(1+ *U*_*C*, 0_/*P*_*C*, 0_)+ 1/(1+ *P*_*C*, 0_/*U*_*C*, 0_) = 1

### Parameter value estimation

The reduced steady state cholesterol concentration can be obtained from a given daily dose of statins or phytosterols/-stanols by applying the reduction model proposed in equation (7). From that reduced concentration, the reduced steady VLDL cholesterol level can be derived by solving equation (2), and subsequently the reduced LDL cholesterol level can be derived by solving equation (3). However, to be applicable in practice, model parameters should be known. In this section all parameters of our model are quantified based on data in the literature.

#### Basic cholesterol model parameters

It is assumed that the liver produces *P*_*C*, 0 _= 1000 mg cholesterol per day [9-12]. Furthermore, we assumed a dietary cholesterol intake of *I_C _= *400 mg/d, of which a fraction of 50% (*f*_*abs*, 0 _= 0.5)[9,13] is taken up in the liver (*U*_*C*, 0 _= 200 mg/d). The same fraction is supposed to be recycled through enterohepatic recycling of cholesterol excreted with bile. The final contribution to liver cholesterol input is assumed to be 70% of produced VLDL cholesterol (*f_back _*= 0.7) [14].

It is assumed that the amount of cholesterol excreted with bile is 1000 mg/d[9,13,15] and consequently, 500 mg/d re-enters the liver. The rate of excretion through the formation of bile salts is 400 mg/d [16]. Concerning the local liver balance, the input is 1000 (produced cholesterol, *P*_*C *,0_) plus 200 (uptake, *U*_*C*, 0_) plus 500 (recycled, (1- *f*_*abs*, 0_)·*k_bile_·C*_0_) plus 700 (back transport, *f_back_·k_VC_V·C*_0_) making a total of 2400 mg/d. The output is 1000 (bile, *k_bile_·C*_0_) plus 400 (bile salts, *k_salts_·C*_0_), and making a total of 2400 mg/d, plus the production of 1000 mg VLDL cholesterol per day (VLDL cholesterol, *k_VC_V·C*_0_). Moreover, as the elimination from the liver is proportional to the production rates of bile salts, cholesterol in bile and VLDL cholesterol, the ratio of these production rates is *k_salts_*: *k_bile_*: *k_VC_V *= 0.4: 1: 1.

From Sahlin *et al*. [17,18] we estimated the free cholesterol content in liver to be 55 nmol/mg microsomal protein. Together with a microsomal protein content of 45 mg/g liver[19] this amounts to 2500 μmol/kg liver which equals 960 mg free cholesterol/kg liver. From this free cholesterol concentration and the daily bile excretion, one can derive *k_bile _= *1000/960 = 1.04, *k_VC_V *= 1.04 and *k_salts _*= 0.416.

Dietschy *et al*. [6] report LDL cholesterol model parameter values in humans. When assuming a subject of 70 kg, these values are *V_max _= *1340 mg/d, *K_M _*= 90 mg/dl, *k_n _*= 5 dl/d and *P_LC _*= 910 mg/d. Based on these values, a steady cholesterol level *LC *= 67 mg/dl results from equation (3).

The VLDL:LDL:HDL cholesterol ratio was estimated to be 1:8:3 [20]. Thus the corresponding VLDL level is 8.4 mg/dl. As the LDL cholesterol production rate is equal to *k_VL_·VC *(equation (3)), *k_VL _= *108 (dl/d). From equation (2) a VLDL cholesterol production of 1020 mg/d can be calculated. Above it is assumed to be 1000 mg/d which shows a consistency error of 2% only.

#### Cholesterol reduction model parameters

From the ratio between the effective clearances, introduced in the basic cholesterol model parameters section, one can derive that the ratio *ρ_k _*in equation (4) is 0.7. The four remaining parameters *R_P, max_*, *S*_*P*, 1/2_, *R_U, max_*, PS_*U*, 1/2 _are unknown and were fitted to optimise their likelihood in comparing modelled LDL cholesterol reduction induced by cholesterol reduction to LDL cholesterol reduction data. Thus, given an estimation of the four cholesterol reduction model parameters, the reduction in steady state cholesterol is calculated, the resulting reduction in VLDL cholesterol is determined from equation (2) and the resulting reduction in LDL cholesterol is determined from equation (3).

To simulate the appropriateness of our model, reduced levels are compared with the experimental data for separate intakes of atorvastatin[21] and phytosterols/-stanols [22]. For this procedure we use for *R_P, max_*, *S*_*P*, 1/2 _data from a recent meta-analysis of Berry *et al*. [21] that shows experimentally determined *in vivo *LDL cholesterol reduction due to atorvastatin dose. For *R_U, max_*, *PS*_*U*, 1/2 _we use data presented in Demonty *et al*. [22] showing experimentally determined *in vivo *LDL cholesterol reduction due to intake of free phytosterols/-stanols, i.e. phytosterols/-stanols not in esterified form. Finally, we simulate reductions after combined intake of atorvastatin and phytosterols/-stanols.

## Results

### Separate intake

#### LDL cholesterol reduction by atorvastatin

We applied equation (7) together with the corresponding VLDL and LDL cholesterol levels equations (2) and (3) to data in Berry *et al*. [21] in a LDL cholesterol reduction model using the model parameters given above. The unknown parameter values in equation (7) were estimated through fitting the maximum reduction *R_P, max _*and the statin dose *S*_*P*, 1/2 _when half maximum reduction is reached.

**Figure 3:**
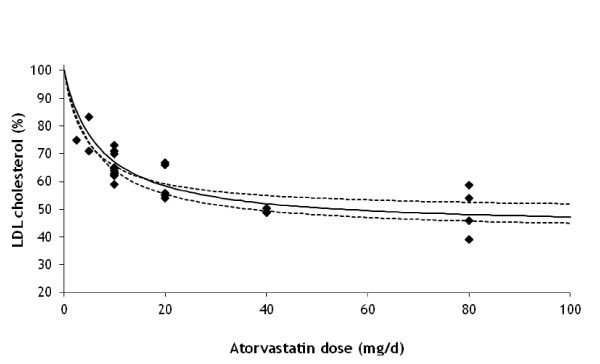
**Simulated reduction (%) in LDL cholesterol after treatment with different doses of atorvastatin**. The solid line shows the fit to the model and symbols represent experimental data from Berry et al. [21] Values for the Michaelis-Menten parameters are: effective maximum LDL cholesterol reduction (*R_P, max_*) = 0.544 and half maximum reduction statin dose (*S*_*P*, 1/2_) = 6.7 mg/d. The dashed lines show the 5% and 95% uncertainty range in reduction obtained by correlated sampling (correlation coefficient *ρ *= 0.88) of *R_P, max _*and *S*_*P*, 1/2 _from their covariance matrix.

Figure 3 shows the comparison of the resulting LDL cholesterol reduction model to the data in Berry *et al*. [21] Parameter values are *R_P, max _*= 0.544 (standard error (SE) = 0.033) and half reduction dose *S*_*P*, 1/2 _= 6.7 (SE = 1.4) mg/d. Given the parsimony of dose levels and scattering of data, a good comparison between the model and the experimental results is obtained (R^2 ^= 0.70).

#### LDL cholesterol reduction by phytosterols/-stanols

We applied equation (7) together with the corresponding VLDL and LDL cholesterol levels equations (2) and (3) to data in Demonty *et al*. [22] in a LDL cholesterol reduction model. The unknown parameter values in equation (7) were estimated through fitting the maximum reduction *R*_*U, max *_and the free phytosterol/-stanol dose *PS*_*U*, 1/2 _when half maximum reduction is reached.

**Figure 4:**
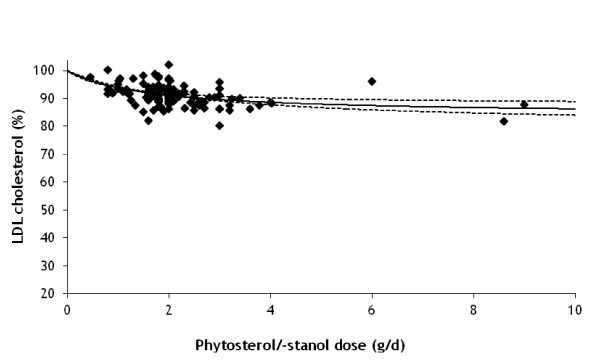
**Simulated reduction (%) in LDL cholesterol after treatment with different doses of phytosterols/-stanols**. The solid line shows the fit to the model and symbols represent experimental data from Demonty et al. [22] Values for the Michaelis-Menten parameters are: effective maximum LDL cholesterol reduction (*R_U, max_*) = 0.221 and half maximum reduction phytosterol/-stanol dose (*PS*_*U*, 1/2_) = 1.78 mg/d. The dashed lines show the 5% and 95% uncertainty range in reduction obtained by correlated sampling (correlation coefficient *ρ *= 0.98) of *R*_*U, max *_and *PS*_*U*, 1/2 _from their covariance matrix.

Figure 4 shows the comparison of the resulting LDL cholesterol reduction model to the data of Demonty *et al*. [22] Parameter values are *R*_*U, max *_= 0.221 (SE = 0.039) and half reduction dose *PS*_*U*, 1/2 _= 1.78 (SE = 0.69) mg/d. The model shows reasonable agreement with the published experimental data (R^2 ^= 0.17).

### Combined intake

#### LDL cholesterol reduction by combined use of atorvastatin and phytosterols/-stanols

**Figure 5:**
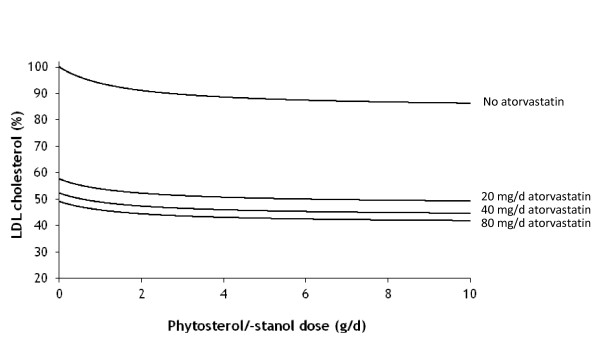
**Simulated reduction (%) in LDL cholesterol after combined treatment with different doses of free phytosterols/-stanols and atorvastatin in humans**. The lines from upper to lower show LDL cholesterol reduction for subjects that are exposed to no atorvastatin, or daily doses of 20, 40 or 80 mg atorvastatin, respectively.

The model is applied to LDL cholesterol reduction due to the combined intake of statins and phytosterols/-stanols. For subjects with a daily intake of 0, 20, 40 and 80 mg atorvastatin, respectively, we show in Figure 5 the total reduction in LDL cholesterol as a function of daily phytosterol/-stanol intake. For the daily recommended intake level of 2 g free phytosterols/-stanols (equivalent to 3.3 g/d phytosterol/-stanol esters), the additional reduction is 4.2% for a daily statin dose of 80 mg, 4.5% for a daily statin dose of 40 mg, 4.8% for a daily statin dose of 20 mg and 7.8% with no statin intake. Thus, the reduction in LDL cholesterol caused by additional phytosterol/-stanol intake decreases with increasing daily atorvastatin dose. However, when considering the additional decrease as a percentage of the LDL cholesterol level already reduced due to statin intake, the additional decrease ranges from 7.8% (no statin intake) to 8.6% (80 mg daily statin dose). Randomised controlled trials in which patients on statin therapy were treated daily with 1.8 to 6 g phytosterol or -stanol esters have shown reductions in the same order of magnitude, i.e. between 6.1% and 10.3% [23-28].

The reduction in enterohepatic recycling contributes for 68%, 58%, 56% and 55% of the total decrease in LDL cholesterol levels at daily statin doses of 0, 20, 40 and 80 mg, respectively. At the same recommended phytosterol/-stanol intake level of 2 g/d, the additional decrease in LDL cholesterol by physterols/-stanols for a daily statin dose of 20 mg (4.8%) is equal to the additional decrease by doubling daily statin dose to 40 mg (5.3%). For a daily statin dose of 40 mg the additional decrease in LDL cholesterol by physterols/-stanols (4.5%) is 30% larger than the additional decrease by doubling daily statin dose to 80 mg (3.2%).

## Discussion

In this paper, a mathematical model is presented that simulates the reductions in LDL cholesterol after separate and combined intake of atorvastatin and phytosterols/-stanols in humans. We demonstrated that a daily intake of 2 g phytosterols/-stanols reduces LDL cholesterol level by about 8% to 9% on top of the reduction resulting from statin use. This level of reduction is consistent with the findings of randomised controlled trials [23-28]. The additional decrease in LDL cholesterol caused by phytosterol/-stanol use at the recommended level of 2 g/d appeared to be similar or even greater than the decrease achieved by doubling the statin dose, a finding that has been observed previously in human trials [23,29]. The reduction in LDL cholesterol level due to phytosterol/-stanol use results from a decrease in the intestinal uptake of dietary cholesterol (additional effect) and a reduction in enterohepatic recycling (multiplicative effect). For daily statin doses of 20 mg or more, the contribution of the enterohepatic recycling reduction is 55% or more. When no statin is used, this contribution is 68%.

Mathematical models provide a valuable means of interpreting experimental data and improving the ability to predict the response to a given treatment. Other modelling studies have focused on cholesterol metabolism, but are merely aimed at answering questions on the cellular level or tend to focus on specific areas of cholesterol metabolism, such as the fluid dynamics of lipid accumulation on the arterial wall or the chemical kinetics of LDL oxidation [30-32].

In the present study, the separate and combined effects of the cholesterol-lowering drug atorvastatin and functional foods with phytosterols/-stanols in humans were modelled. Yet, this model can easily be applied to other statins and similar acting (functional) foods as well. Products with soluble dietary fibres, for example, are also known to lower total and LDL cholesterol by reducing the intestinal (re)absorption of cholesterol and bile acids, although they work by a different mechanism as phytosterols/-stanols [4,33,34]. Moreover, individuals' specific reductions in total and LDL cholesterol can be predicted, based on certain genetic variants. For example, the ratio of cholesterol synthesis to cholesterol absorption varies between individuals and is an important determinant for the cholesterol pool size [35]. Also mutations in the LDL receptor gene causing familial hypercholesterolaemia can be modelled by varying the parameter *V_max_*.

There are a few possible directions for improving our model. First, the model could be extended by including the up- and down regulatory mechanisms involving the LDL receptors. Nonetheless, since we assumed that the clearing of (V)LDL cholesterol from the blood follows Michaelis-Menten kinetics, we implicitly included receptor-mediated uptake in the model. Also other regulatory control pathways were disregarded, such as the existence of a hepatic cholesteryl ester pool that might be involved in cholesterol homeostasis and the regulatory loop in the synthesis of LDL receptors [30]. Another extension would include reverse cholesterol transport mediated by HDL [20]. Moreover, the proposed model assumes that the reducing effects of statins and functional foods are independent of each other. Although this is likely the case for the combination of phytosterols/-stanols and statins,[22,24,36,37] it is uncertain whether this applies for other food-drug combinations. It has, for example, been proposed that soluble dietary fibres reduce the intestinal uptake of statins [38,39]. Our model should be extended to include such an interaction.

In conclusion, we proposed a simplified mathematical model to simulate the reduction in LDL cholesterol after separate and combined intake of statins and functional foods acting on intestinal (re)absorption of cholesterol or bile acids in humans. In future work, this model can be extended to include more complex (regulatory) mechanisms.

## Competing interests

The authors declare that they have no competing interests.

## Authors' contributions

SE and JvE developed, tested and validated the model, and drafted the first version of the manuscript. Subsequent versions of the report were written by SE and JvE with input and critical revisions by all authors. All authors have read and approved the final manuscript as submitted.

## Appendix 1

### Steady VLDL cholesterol concentration

The mass balance equation (2) can be rewritten as a quadratic equation in *VC*:

$$k_{VL}\cdot VC^{2}+\left(V_{max}+k_{VL}\cdot K_{M}-k_{VC}V\cdot C\right)\cdot VC-K_{M}\cdot k_{VC}V\cdot C=0$$

Here, we suppress the dependencies on statin administration *S *and dietary phytosterol/-stanol intake *PS*.

A quadratic equation has two solutions, but the only physicochemical relevant solution for which the VLDL cholesterol concentration is non-negative is:

$$\begin{array}{ccc} VC & =\frac{1}{2k_{VL}}\left(k_{VC}V\cdot C-k_{VL}\cdot K_{M}-V_{max}\right) & \\ & \left(+\sqrt{{\left(k_{VC}V\cdot C-k_{VL}\cdot K_{M}-V_{max}\right)}^{2}+4k_{VL}\cdot K_{M}\cdot k_{VC}V\cdot C}\right) \end{array}$$

### Steady LDL cholesterol concentration

Like for VLDL cholesterol, the mass balance equation (3) can be rewritten as:

$$k_{n}\cdot LC^{2}+\left(V_{max}+k_{n}\cdot K_{M}-k_{VL}\cdot VC\right)\cdot LC-K_{M}\cdot k_{VL}\cdot VC=0$$

with as solution:

$$\begin{array}{ccc} LC & =\frac{1}{2k_{n}}\left(k_{VL}\cdot VC-k_{n}\cdot K_{M}-V_{max}\right) & \\ & \left(+\sqrt{{\left(k_{VL}\cdot VC-k_{n}\cdot K_{M}-V_{max}\right)}^{2}+4k_{n}\cdot K_{M}\cdot k_{VL}\cdot VC}\right) \end{array}$$

## Appendix 2

### Reduction in steady state cholesterol

The reduction in steady state cholesterol level is:

$$\begin{array}{ccc} R_{C}\left(S,PS\right) & =\frac{C\left(S,PS\right)}{C_{0}} & \\ & =\frac{\left(P_{C}\left(S\right)+f_{abs}\left(PS\right)\cdot I_{C}\right)∕\left(\left(1-f_{back}\right)\cdot k_{VC}V+\left(1-f_{abs}\left(PS\right)\right)\cdot k_{bile}+k_{salts}\right)}{\left(P_{C,0}+f_{abs,0}\cdot I_{C}\right)∕\left(\left(1-f_{back}\right)\cdot k_{VC}V+\left(1-f_{abs,0}\right)\cdot k_{bile}+k_{salts}\right)}\\ & =\frac{\left(1-f_{back}\right)\cdot k_{VC}V+\left(1-f_{abs,0}\right)\cdot k_{bile}+k_{salts}}{\left(1-f_{back}\right)\cdot k_{VC}V+\left(1-f_{abs}\left(PS\right)\right)\cdot k_{bile}+k_{salts}}\cdot \frac{P_{C}\left(S\right)+f_{abs}\left(PS\right)\cdot I_{C}}{P_{C,0}+f_{abs,0}\cdot I_{C}}\\ & =\frac{\rho_{k}+1-f_{abs,0}}{\rho_{k}+1-f_{abs}\left(PS\right)}\cdot \left(\frac{P_{C}\left(S\right)}{P_{C,0}+U_{C,0}}+\frac{f_{abs}\left(PS\right)\cdot I_{C}}{P_{C,0}+f_{abs,0}\cdot I_{C}}\right)\\ & =\frac{\rho_{k}+1-f_{abs,0}}{\rho_{k}+1-f_{abs}\left(PS\right)}\cdot \left(\frac{R_{P}\left(S\right)}{1+U_{C,0}∕P_{C,0}}+\frac{R_{U}\left(PS\right)}{1+P_{C,0}∕U_{C,0}}\right) \end{array}$$

In the third line, the ratio of clearance rates *ρ_k _*= ((1-*f_back_*)·*k_VC_V *+ *k_salts_*)/*k_bile _*is introduced. In this line also one instance of *f*_*abs*, 0_·*I_C _*is substituted by *U*_*C*, 0_. In the fourth line the definition of production reduction and, after dividing out intake *I_C_*, of uptake reduction is substituted.

## References

[B1] Randomised trial of cholesterol lowering in 4444 patients with coronary heart disease: the Scandinavian Simvastatin Survival Study (4S) Lancet 1994 344 8934 1383 1389 7968073

[B2] Baigent C Keech A Kearney PM Blackwell L Buck G Pollicino C Kirby A Sourjina T Peto R Collins R Simes R Efficacy and safety of cholesterol-lowering treatment: prospective meta-analysis of data from 90,056 participants in 14 randomised trials of statins Lancet 2005 366 9493 1267 78 16214597 10.1016/S0140-6736(05)67394-1

[B3] Amarenco P Labreuche J Lavalle P Touboul P Statins in stroke prevention and carotid atherosclerosis: systematic review and up-to-date meta-analysis Stroke 2004 35 12 2902 2909 10.1161/01.STR.0000147965.52712.fa 15514180

[B4] Plat J Mensink RP Plant stanol and sterol esters in the control of blood cholesterol levels: mechanism and safety aspects Am J Cardiol 2005 96 1A 15D 22D 15992511 10.1016/j.amjcard.2005.03.015

[B5] Marinangeli CP Varady KA Jones PJ Plant sterols combined with exercise for the treatment of hypercholesterolemia: overview of independent and synergistic mechanisms of action J Nutr Biochem 2006 17 4 217 24 10.1016/j.jnutbio.2005.09.003 16410048

[B6] Dietschy JM Turley SD Spady DK Role of liver in the maintenance of cholesterol and low density lipoprotein homeostasis in different animal species, including humans J Lipid Res 1993 34 10 1637 59 8245716

[B7] Shum YY Huang N Walter G Black A Sekerke C Chang T Whitfield LR Development, validation, and interlaboratory comparison of an HMG-CoA reductase inhibition assay for quantitation of atorvastatin in plasma matrices Ther Drug Monit 1998 20 1 41 49 10.1097/00007691-199802000-00008 9485553

[B8] Davis H Altmann S Niemann-Pick C1 Like 1 (NPC1L1) an intestinal sterol transporter Biochim Biophys Acta 2009 1791 7 679 683 10.1016/j.bbalip.2009.01.002 19272334

[B9] McNamara DJ Effects of fat-modified diets on cholesterol and lipoprotein metabolism Annu Rev Nutr 1987 7 273 290 10.1146/annurev.nu.07.070187.001421 3300736

[B10] Rajaratnam RA Gylling H Miettinen TA Cholesterol absorption, synthesis, and fecal output in postmenopausal women with and without coronary artery disease Arterioscler Thromb Vasc Biol 2001 21 10 1650 1655 10.1161/hq1001.097019 11597940

[B11] Insel P Ross D McMahon K Bernstein M (eds) Nutrition 2011 Jones and Bartlett Publishers, Sudbury, MA

[B12] Stipanuk MH (ed) Biochemical and physiological aspects of human nutrition. Lipid metabolism-Synthesis and oxidation 2000 W.B. Saunders Co., Philadelphia, USA

[B13] van der Velde A Brufau G Groen A Transintestinal cholesterol efflux Curr Opin Lipidol 2010 21 3 167 171 10.1097/MOL.0b013e3283395e45 20410820

[B14] Demirezen EM Barlas Y A simulation model for blood cholesterol dynamics and related disorders 2009 2011 12 July 1 38

[B15] Phillips GB The lipid composition of human bile Biochim Biophys Acta 1960 41 361 363 10.1016/0006-3002(60)90026-3 14432614

[B16] Bisschop P Bandsma RHJ Stellaard F Harmsel A Meijer A Sauerwein H Kuipers F Romijn J Low-fat, high-carbohydrate and high-fat, low-carbohydrate diets decrease primary bile acid synthesis in humans Am J Clin Nutr 2004 79 4 570 576 15051599 10.1093/ajcn/79.4.570

[B17] Sahlin S Granstrm L Gustafsson U Sthlberg D Backman L Einarsson K Hepatic esterification rate of cholesterol and biliary lipids in human obesity J Lipid Res 1994 35 3 484 490 8014583

[B18] Sahlin S Sthlberg D Einarsson K Cholesterol metabolism in liver and gallbladder mucosa of patients with cholesterolosis Hepatology 1995 21 5 1269 1275 7737633

[B19] Houston JB Utility of in vitro drug metabolism data in predicting in vivo metabolic clearance Biochem Pharmacol 1994 47 9 1469 1479 10.1016/0006-2952(94)90520-7 8185657

[B20] Kumar PJ Clark ML Clinical medicine - Diabetes Mellitus and other disorders of metabolism W.B. Saunders 2002

[B21] Berry DA Berry SM McKellar J Pearson TA Comparison of the dose-response relationships of 2 lipid-lowering agents: a Bayesian meta-analysis Am Heart J 2003 145 6 1036 1045 10.1016/S0002-8703(03)00106-6 12796760

[B22] Demonty I Ras RT van der Knaap HC Duchateau GS Meijer L Zock PL Geleijnse JM Trautwein EA Continuous dose-response relationship of the LDL-cholesterol-lowering effect of phytosterol intake J Nutr 2009 139 2 271 84 19091798 10.3945/jn.108.095125

[B23] Blair SN Capuzzi DM Gottlieb SO Nguyen T Morgan JM Cater NB Incremental reduction of serum total cholesterol and low-density lipoprotein cholesterol with the addition of plant stanol ester-containing spread to statin therapy Am J Cardiol 2000 86 1 46 52 10.1016/S0002-9149(00)00976-0 10867091

[B24] Simons LA Additive effect of plant sterol-ester margarine and cerivastatin in lowering low-density lipoprotein cholesterol in primary hypercholesterolemia Am J Cardiol 2002 90 7 737 40 10.1016/S0002-9149(02)02600-0 12356387

[B25] Richter WO Treatment of severe hypercholesterolemia with a combination of beta-sitosterol and lovastatin Curr Ther Res 1996 57 7 497 505 497 10.1016/S0011-393X(96)80059-2

[B26] Castro Cabezas M de Vries JH Van Oostrom AJ Iestra J van Staveren WA Effects of a stanol-enriched diet on plasma cholesterol and triglycerides in patients treated with statins J Am Diet Assoc 2006 106 10 1564 9 10.1016/j.jada.2006.07.009 17000189

[B27] Goldberg AC E OR Jr Bateman JH Schimmoeller L McPherson TB Spilburg CA Effect of plant stanol tablets on low-density lipoprotein cholesterol lowering in patients on statin drugs Am J Cardiol 2006 97 3 376 9 10.1016/j.amjcard.2005.08.056 16442399

[B28] De Jong A Plat J Bast A Godschalk RW Basu S Mensink RP Effects of plant sterol and stanol ester consumption on lipid metabolism, antioxidant status and markers of oxidative stress, endothelial function and low-grade inflammation in patients on current statin treatment Eur J Clin Nutr 2008 62 263 73 10.1038/sj.ejcn.1602733 17487211

[B29] Katan MB Grundy SM Jones P Law M Miettinen T Paoletti R Efficacy and safety of plant stanols and sterols in the management of blood cholesterol levels Mayo Clin Proc 2003 78 8 965 78 10.4065/78.8.965 12911045

[B30] August E Parker KH Barahona M A dynamical model of lipoprotein metabolism Bull Math Biol 2007 69 4 1233 1254 10.1007/s11538-006-9132-6 17334872

[B31] Chun PW Espinosa AJ Lee CW Shireman RB Brumbaugh EE Low density lipoprotein receptor regulation. Kinetic models Biophys Chem 1985 21 3-4 185 209 10.1016/0301-4622(85)80005-3 2985137

[B32] Wattis JAD O'Malley B Blackburn H Pickersgill L Panovska J Byrne HM Jackson KG Mathematical model for low density lipoprotein (LDL) endocytosis by hepatocytes Bull Math Biol 2008 70 8 2303 2333 10.1007/s11538-008-9347-9 18716843 PMC2784520

[B33] Theuwissen E Mensink RP Water-soluble dietary fibers and cardiovascular disease Physiol Behav 2008 94 2 285 92 10.1016/j.physbeh.2008.01.001 18302966

[B34] Eussen S Klungel O Garssen J Verhagen H van Kranen H van Loveren H Rompelberg C Support of drug therapy using functional foods and dietary supplements: focus on statin therapy Br J Nutr 2010 103 9 1260 1277 10.1017/S0007114509993230 20196891

[B35] Gylling H Miettinen TA Baseline intestinal absorption and synthesis of cholesterol regulate its response to hypolipidaemic treatments in coronary patients Atherosclerosis 2002 160 2 477 81 10.1016/S0021-9150(01)00608-6 11849674

[B36] Eussen S de Jong N Rompelberg C Garssen J Verschuren W Klungel O Dose-dependent cholesterol-lowering effects of phytosterol/phytostanol-enriched margarine in statin users and statin non-users under free-living conditions Public Health Nutr 2011 1 10 10.1017/S1368980011000164 21356148

[B37] de Jong N Zuur A Wolfs MC Wendel-Vos GC van Raaij JM Schuit AJ Exposure and effectiveness of phytosterol/-stanol-enriched margarines Eur J Clin Nutr 2007 61 1407 15 10.1038/sj.ejcn.1602660 17299474

[B38] Richter WO Jacob BG Schwandt P Interaction between fibre and lovastatin Lancet 1991 338 8768 706 1679514 10.1016/0140-6736(91)91291-2

[B39] Eussen SRBM Rompelberg CJM Andersson K Klungel O Hellstrand P Oste R van Kranen H Garssen J Simultaneous intake of oat bran and atorvastatin reduces their efficacy to lower lipid levels and atherosclerosis in LDLr-/- mice Pharmacological research 2011 64 1 36 43 10.1016/j.phrs.2011.02.008 21371558

